# The association of frailty with cognition outcomes in SPRINT‐MIND

**DOI:** 10.1002/alz.095414

**Published:** 2025-01-09

**Authors:** Kathryn E. Callahan, Byron C Jaeger, Stephen R. Rapp, Nicholas M. Pajewski, Jeff D. Williamson

**Affiliations:** ^1^ Wake Forest University School of Medicine, Winston‐Salem, NC USA

## Abstract

**Background:**

Frailty may moderate efficacy of interventions for Alzheimer’s disease (AD) and related dementias (ADRD), with higher efficacy at lower neuropathology burden in frailty. Using data from the Systolic Blood Pressure Intervention Trial (SPRINT) with extended post‐trial follow‐up, we assessed whether frailty moderated the effect of intensive blood pressure (BP) control and subsequent cognitive outcomes.

**Methods:**

Primary outcomes were probable dementia (PD) and mild cognitive impairment (MCI). Intensive and standard BP control targeted systolic BP < 120 and < 140 mmHg, respectively. Frailty index (FI) was calculated using a deficit‐accumulation model. We tested for heterogeneity in the effect of intensive BP control among participants who were non‐frail (FI ≤ 0.10), pre‐frail (0.10 < FI ≤ 0.21), and frail (FI > 0.21) using Cox proportional hazards, accounting for differential baseline hazard by study site. Analyses were replicated in subgroups defined by age, sex, and race.

**Results:**

Of the 9361 participants in the SPRINT trial, 8541 consented to follow‐up for PD/MCI and had calculable baseline FI. Over 54568 person‐years, 539 PD and 809 MCI events occurred. Participants categorized as non‐frail (N = 1412) were younger and more likely to be male versus pre‐frail (N = 4430) and frail (N = 2699) participants. Intensive versus standard BP control exhibited a heterogeneous effect for PD risk reduction (p = 0.03), with hazard ratios (HRs) ranging from 0.70 to 1.10. Among subgroups based on age, sex, and race, PD HRs ranged from 0.59 to 1.00. For MCI, heterogeneity was not detected (p = 0.23), and HRs ranged from 0.70 to 1.00. No evidence of harm (i.e., HR > 1) was detected.

**Conclusions:**

Heterogeneity based on frailty in the effect of BP control may impact subsequent cognitive outcomes. Future studies with cognitive outcomes should consider stratifying by frailty status.

